# The breadth of bread: unearthing genomic trade-offs in 355 wheat accessions

**DOI:** 10.1093/plcell/koad235

**Published:** 2023-09-12

**Authors:** Marco Bürger

**Affiliations:** Assistant Features Editor, The Plant Cell, American Society of Plant Biologists; Plant Biology Laboratory, Salk Institute for Biological Studies, La Jolla, CA 92037, USA

Wheat is a globally important staple food and cereal grain, with a world production of more than 750 million tons. It was first cultivated around 8,000 years ago in the area of the Fertile Crescent. As ancient farmers began selectively breeding wheat for desirable traits, they laid the foundation for what would eventually become landraces. These landraces are traditional varieties of crops tailored to withstand local stresses while delivering intermediate yields under low-input farming conditions. The transition from these traditional varieties to elite cultivars signifies a human-directed evolutionary process to achieve better yields, quality, and overall fitness (Liu et al. 2019). However, by relying on a narrow set of founders for breeding, there is a reduction in genetic diversity, called the bottleneck effect (Lopes et al. 2015). A limited genetic base can mean that the resulting cultivars might share common vulnerabilities, for example, disease susceptibility. For this reason, the preservation and study of landraces have become increasingly important, and while modern cultivars offer high yields and uniformity that meet current agricultural demands, the genetic diversity present in landraces ensures that crops can be resilient and adaptable in the face of future challenges (Milner et al. 2019).

To capture the vast spectrum of genetic information, in this issue of *The Plant Cell*, **Jianqing Niu, Shengwei Ma, Shusong Zheng, Chi Zhang, and colleagues** (Niu et al. 2023) embarked on a massive sequencing project of both wheat landraces and modern cultivars. The researchers sequenced the genomes of 355 wheat accessions to identify genomic regions targeted by modern wheat breeding. They sampled 175 cultivars and 180 diverse landraces from 14 countries and identified 76.9 million single-nucleotide polymorphisms (SNPs) from the whole-genome sequencing data, with significant variations found in genes affecting plant traits like height, vernalization response, and disease resistance. To understand the population structure among the 355 common wheat accessions, the authors used 304,744 SNPs selected based on linkage disequilibrium to construct genetic assignment analysis and perform principal component analysis. They calculated nucleotide diversity and differentiation across the subpopulations of landraces, Chinese cultivars, and U.S. cultivars. While most cultivars from China and the United States were distinctly separate from other accessions, a mixed subgroup suggested genetic material exchange between the two countries. Landraces showed higher genetic diversity compared with the cultivars, confirming that a bottleneck effect in modern wheat breeding does exist.

Niu and co-workers then assessed 21 agronomic traits over 3 years, revealing that both Chinese and U.S. cultivars exhibited similar directional changes in specific wheat traits, but the magnitude of these changes was more pronounced in the Chinese cultivars (see Fig.). When they performed genome-wide association studies on these traits, they identified 5,931 marker–trait associations assigned to 207 loci with known significant thresholds. Six of these associated loci were found in genomic regions containing already recognized genes like *Reduced height-2* (*Rht2*), *FRIZZY PANICLE* (*WFZP*), and *PHOTOPERIOD 1* (*PPD1*), and interestingly, many of these candidate genes have rice homologs that are responsible for similar traits. Many of the identified beneficial alleles were more common in the modern cultivars from both countries compared with the landraces, suggesting that modern breeding practices in both China and the United States have increased the frequency of these beneficial gene versions. Last, the authors explored genetic loci that underlie wheat morphological features. By comparing cultivars to landrace populations in both countries, they identified selection signatures associated with wheat improvement. They found 2,037 and 1,866 selective sweeps in Chinese and U.S. cultivars, respectively, covering substantial portions of the wheat genome. These regions contained several genes with known functions related to plant architecture, growth period, resistance, and grain quality, with some genes showing evidence of selection only in one country but not the other, emphasizing diverse breeding focuses in the two regions.

**Figure.**
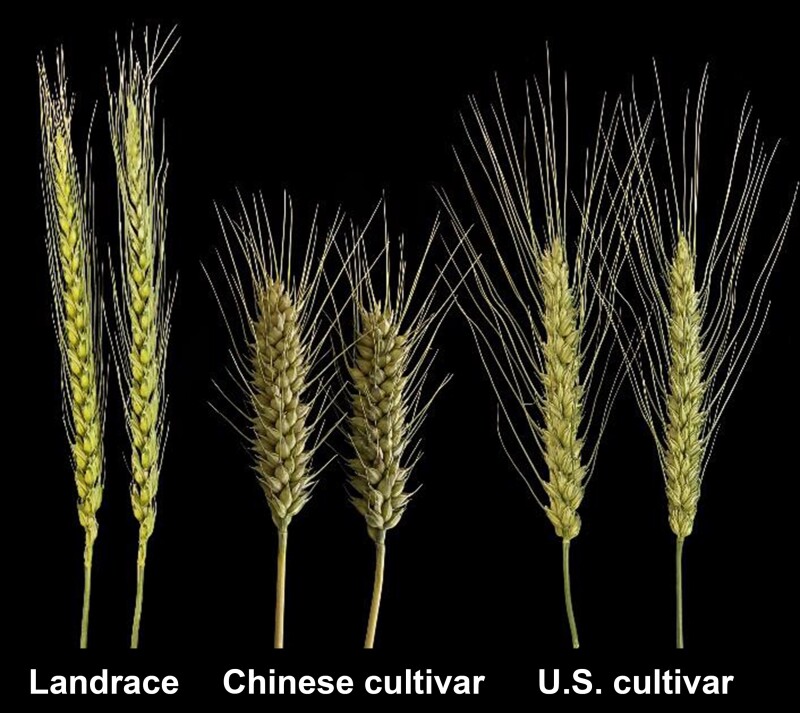
Phenotypes of wheat spikes from the landrace PI 321967, the Chinese cultivar Jimai 20, and the U.S. cultivar Ripper. Adapted from Niu et al. 2023, Figure 2H.

The work by Niu et al. (2023) not only sheds light on the genetic changes that have occurred in wheat breeding but also underscores the importance of preserving and studying landraces. The region-specific breeding targets identified in this study will be a great resource for future wheat improvement. By understanding these genetic nuances, breeders can harness the rich genetic diversity of landraces, ensuring a resilient future for one of the world's most crucial staple foods.

## Dive Curated Terms

The following phenotypic, genotypic, and functional terms are of significance to the work described in this paper:

PPD1 Gramene: AT4G14713
PPD1 Araport: AT4G14713
